# Promoting EF With Preschool Interventions: Lessons Learned From 15 Years of Conducting Large-Scale Studies

**DOI:** 10.3389/fpsyg.2021.640702

**Published:** 2021-06-24

**Authors:** Shira Mattera, Natalia M. Rojas, Pamela A. Morris, Karen Bierman

**Affiliations:** ^1^MDRC, New York, NY, United States; ^2^Department of Population Health, New York University School of Medicine, New York, NY, United States; ^3^Department of Applied Psychology, New York University, New York, NY, United States; ^4^Department of Psychology, Pennsylvania State University, State College, PA, United States

**Keywords:** executive function, preschool, interventions, school readiness, academic achievement

## Abstract

In the past two decades, a growing number of early childhood interventions that aim to improve school readiness have also targeted children's executive function (EF), building on the theory that promoting EF skills in preschool may play a key role in reducing the substantial gaps in school readiness and later achievement associated with family income. Despite the expansion of school readiness interventions across preschool, research evidence is mixed regarding what works to promote EF development and the impact of these interventions on children's EF skills, and subsequently, their academic and behavioral outcomes. This paper reviews four intervention approaches designed to support school readiness that may also improve children's EF skills by: (a) encouraging adaptive classroom behaviors, (b) improving social-emotional learning, (c) promoting play and direct training of EF skills, and (d) improving cognitive skills related to EF. We describe program effects from rigorous trials testing these approaches, including summarizing the takeaways from four large-scale intervention research studies conducted by the authors, involving over 5,000 children. We conclude by exploring open questions for the field and future directions for research and intervention program development and refinement.

## Introduction

Children's executive function (EF) has been seen as a potentially potent target of early childhood interventions over the past 15 to 20 years. EF reflect a child's capacity to exert self-regulatory cognitive processes in order to achieve a goal (Garon et al., 2008). These overarching functions help a child manage their cognitive, social, emotional, and behavioral responses, especially in situations requiring novel problem-solving. EFs are used for difficult tasks that evoke effortful self-monitoring, instead of automated responses (Hughes and Graham, 2002). Whereas crystallized cognition encompasses basic knowledge and facts, EF represents while fluid cognition and reflects cognitive-based processes that oversee, or execute, the use of behavioral and cognitive strategies (Blair, 2006).

Children living in poverty often experience environments that can hamper the development of EF skills (Noble et al., 2007), which lay the groundwork for the development of mental systems that support self-regulation and adaptive learning behaviors (Blair, 2002). EF has been identified as a promising lever for the preschool years because those skills: (1) normatively undergo significant growth during the preschool years, (2) are considered malleable, and (3) are associated with improved social-emotional and academic outcomes. The hope was that if existing school-readiness-focused preschool interventions could also increase the speed of growth of EF skills, they may initiate a cascade of positive subsequent social, behavioral, and academic benefits.

The focus on EF as a target of intervention in the preschool years emerged from two parallel sets of research in the early 2000s. Neuro-developmental work at the time was extending the study of EF in adults down to the earlier years, spurring work that examined the development and importance of EF in young children. A growing body of literature at the time pointed to evidence that EF skills grow and change in a dramatic fashion during the preschool years (e.g., Hughes, 1998; Carlson, 2005; Garon et al., 2008). Correlational research on children's preschool skills suggested that EF underlay a child's ability to self-regulate which, in turn, was associated with more adaptive approaches to learning in the classroom, improved school readiness, and stronger academic skills documented through the sixth grade (e.g., Blair, 2002; Raver, 2002; Espy et al., 2004; McClelland et al., 2006).

At the same time, federal funding priorities in the United States supported a burst in translational research aimed to test preschool programs that could improve school readiness, many with an emphasis on promoting self-regulation as a key outcome (e.g., Webster-Stratton, 1994; Lynch et al., 2004; Domitrovich et al., 2007). Self-regulation has been conceptualized as a larger umbrella term that, in addition to EF, includes regulation of children's behavior, cognition, and emotion (e.g., Raver, 2002). Interestingly, the early childhood programs that also aimed to promote children's self-regulation varied in their conceptual foundations and approaches to improving self regulation. Some of these programs trained teachers in behavior management strategies to encourage adaptive classroom behaviors (e.g., Incredible Years, Webster-Stratton et al., 2001). Others used a curriculum as a vehicle for directly improving social-emotional learning (e.g., Preschool PATHS, Lynch et al., 2004; Domitrovich et al., 2007). Another approach promoted play and direct training of EF skills (e.g., Tools of the Mind, Bodrova and Leong, 2006; Red Light, Purple Light; McClelland et al., 2007). A final approach improved cognitive skills related to EF (e.g., Building Blocks, Clements and Sarama, 2008).

These two sets of research came together to inform a subsequent set of randomized trials of school readiness programs that included explicit measures of EF as well as behavioral measures of self-regulation, with potential to shed light on the effects of these programs on EF and related behavioral and emotional skills and behaviors (e.g., Head Start REDI, Chicago School Readiness Project, Foundations of Learning, Head Start CARES, Making Pre-K Count). Mixed findings emerged from these studies; it was not clear whether curricula that targeted school readiness domains such as cognition (reading, math), social-emotional development, and self-regulation skills significantly improved children's EF skills, and subsequently, children's academic skills. Experts suggested several reasons why there may not have been consistent observable impacts of school readiness interventions on children's EF outcomes, including definitional and measurement issues or curricula that are too complex or hard to implement (Jacob and Parkinson, 2015). While a number of meta-analyses have examined the effects of programs designed specifically to directly improve EF (e.g., Takacs and Kassai, 2019; Pauli-Pott et al., 2020; Scionti et al., 2020), few discussions have synthesized and delved into the effects specifically of these school-readiness-focused programs.

Building from our 15 years of experience conducting school-based RCTs, in this paper we describe different approaches school readiness-focused programs have taken to improve EF in early childhood, lay out the evidence base around those approaches including findings from large-scale preschool intervention studies that also targeted EF as an outcome of interest, and explore future directions for research and intervention program development and refinement.

### Large-Scale Studies of Preschool Interventions Targeting EF

Four large-scale, rigorous studies of five school readiness-focused preschool programs provide a foundation for the authors' reflections on EF. These cluster-randomized controlled trials span 23 cities and 268 preschool sites or centers and include over 5,000 4-year-old children (see Tables 1–4 for more detail about each study):

**Table 1:** Background characteristics of the behavioral-focused studies.

**Study characteristics**	**The Incredible Years Webster-Stratton et al. (2004)**	**CSRP Raver et al., 2009, 2011**	**Foundations of Learning–Newark Morris et al., 2013**	**Foundations of Learning–Chicago Morris et al., 2013**	**The Incredible Years–CARES study Mattera et al., 2013; Morris et al., 2014**
Design overview	Families were assigned at random to one of six conditions: parent training alone (PT); child training alone (CT); parent training plus teacher training (PT + TT); child training plus teacher training (CT + TT); parent and child training combined with teacher training (PT + CT + TT); and a waiting list control group	Paired randomized trial, Head Start centers were randomly assigned to the intervention or business as usual control condition	Randomized trial, Head Start centers were randomly assigned to the FOL intervention or business as usual control condition	Randomized trial; Head Start, community-based centers, and public schools were randomly assigned to the FOL intervention or business as usual control condition	Cluster randomized trial; centers randomly assigned to one of three curricula (IY, PP, or TM) or business-as usual control condition
Study location	Washington state	Chicago, Il	Newark, NJ	Chicago, Il	17 Head Start grantees located in 10 states across the United States
Sample size	159 children and families; 8 clinicians	18 sites; 90 Head Start classrooms; 87 teachers; 543 children	51 sites; 51 classrooms/teachers; 531 children	20 sites; 40 classrooms/teachers; 307 children	104 sites; 307 classrooms; 2,114 (total sample) and 702 incredible years program group children
Child sample demographics	90% boys, 79% Euro-American, with a mean age of 70.99 months	67% African-American, 25% Hispanic, and 3% white, with a mean age of 49.4 months	68% African-American, 28% Hispanic, and 2% White, with a mean age of 4.1 years	43% African-American, 35% Hispanic, and 10% White, with mean age 4.4 years	43% were Hispanic, 33% African-American, and 16% white, with a mean age 4.4 years
**Intervention characteristics**					
Duration of implementation	1 year	1 year	1 year	1 year	1 year
Amount of training and coaching	Teachers: 4 days of training	5 training sessions (30 h total); weekly coaching visits	5 training sessions; weekly coaching visits	5 training sessions; weekly coaching visits	6 training sessions; weekly coaching visits
**Measures**					
Teacher practice	Classroom atmosphere measure (OA)				Adapted teacher style rating scale (TSRS) (OA)
Classroom climate	MOOSES (OA); Teacher coder impressions inventory (OA)	CLASS (OA); ECERS-R (OA)	CLASS (OA)		CLASS (OA)
Executive function and behavioral regulation	Teacher assessment of school behavior (TR); MOOSES (OA); social health profile (AR)	Behavioral problem index (TR); caregiver-teacher report form (TR); penn interactive peer play scale (OA); PSRA (AR); balance beam (DA); pencil tap (DA)	Behavioral problem index (TR); caregiver-teacher report form (TR); cooper-farran behavioral ratings scale (TR)	inCLASS (OA); Behavioral problem index (TR); caregiver-teacher report form (TR); cooper-farran behavioral ratings scale (TR); head-to-toes (DA); pencil tap (DA); gift wrap (TR); preschool self-regulation interviewer assessment (AR)	Head-to-Toes (DA); pencil tap (DA); behavioral problem index (TR)
Emotion knowledge and social problem-solving skills	Perceived competence scale for young children (TR)	Toy wrap (DA); toy wait (DA); snack delay (DA); tongue task (DA)	Student-Teacher relationships scale (TR)	Challenging situations (DA);	Facial emotions identification tasks (DA); emotion situation tasks (DA); challenging situations task (DA)
Learning and social behaviors	DPIS (DA)		inCLASS (OA); positive behavior scale (TR)	Positive behavior scale (TR)	Social skills rating system (TR)
Academic outcomes		PreLAS (DA); peabody picture vocabulary test (DA); early math skills (DA)	Academic rating scale (TR)	WJ-III Letter-word identification (DA); WJ-III applied problems (DA); peabody picture vocabulary test (DA); academic rating scale (TR)	WJ-III Letter-word identification (DA); WJ-III applied problems (DA); EOWPVT (DA); academic rating scale (TR)

*DA, direct child assessment; TR, teacher-report; OA, observational assessment; AR, assessor-report*.

**Table 2:** Background characteristics of the social-emotional learning studies.

**Study characteristics**	**Preschool PATHS Domitrovich et al., 2007**	**Preschool PATHS–CARES study Mattera et al., 2013; Morris et al., 2014**	**Head Start REDI Bierman et al., 2008; Domitrovich et al., 2009**
Design overview	Mixed-block randomized trial; classrooms were randomly assigned to either preschool PATHS or business-as-usual control condition	Cluster randomized trial; centers randomly assigned to one of three curricula (IY, PP, or TM) or business-as usual control condition	Cluster randomized trial; centers randomly assigned to intervention or business-as-usual condition
Sample location	Central Pennsylvania	17 head start grantees located in 10 states across the United States	Pennsylvania
Sample size	2 head start programs; 20 classrooms/teachers; 246 children	307 classrooms; 2,114 (total sample) and 669 preschool Paths program group children	44 head start classrooms/teachers; 356 children
Child sample demographics	47% African-American, 38% European-American, and 20% were Hispanic with a mean age of 51.40 months.	43% were Hispanic, 33% African-American, and 16% White, with a mean age 4.49 years	356 children: 17% Hispanic, 25% African-American
**Intervention characteristics**			
Duration of implementation	1 year	1 year	1 year
Amount of training and coaching	3 days; monthly coaching visits	4 training sessions; weekly coaching visits	Training sessions and weekly coaching visits
**Measures**			
Teacher practice		Adapted teacher style rating scale (TSRS) (OA)	
Classroom climate		CLASS (OA)	
Executive function and behavioral regulation	Day/Night task (DA); Attention sustained subtest from the leiter-revised assessment battery (DA); Problem behavior scale of the PKBS (TR)	Head-to-Toes (DA); pencil Tap (DA); behavioral problem index (TR)	ADHD rating scale (TR)
Emotion knowledge and social problem-solving skills	Recognition of emotions concepts from KEI (DA); assessment of children's emotions scale (DA); Denham puppet interview (DA); challenging situations (DA)	Facial emotions identification tasks (DA); emotion situation tasks (DA); challenging situations task (DA)	Children's emotion skills (DA); emotion recognition questionnaire (DA); challenging situations tasks (DA)
Learning and social behaviors	Social skills scale of the PKBS (TR)	Social skills rating system (TR)	Social competence scale (TR); Teacher observation of child adaptation-revised (TR); learning engagement (TR)
Academic outcomes		WJ-III Letter-word identification (DA); WJ-III applied problems (DA); EOWPVT (DA); academic rating scale (TR)	EOWPVT (DA); Grammatical understanding subtest of the test of language development (DA); Test of preschool early literacy (DA)

*DA, direct child assessment; TR, teacher-report; OA, observational assessment; AR, assessor-report*.

**Table 3:** Background characteristics of the promoting play and direct training studies.

**Study characteristics**	**Tools of the Mind Diamond et al., 2007**	**Tools of the Mind Clements et al., 2020**	**Tools of the Mind Lonigan and Phillips, 2012**	**Tools of the Mind Farran and Wilson, 2014**	**Tools of the Mind–Play–CARES study Mattera et al., 2013; Morris et al., 2014**	**Tools of the Mind Solomon et al., 2018**	**Red Light/Purple Light McClelland et al., 2019**
Design overview	Children were randomly assigned to classrooms with tools of the mind or literacy in a balanced way curriculum	Three-armed cluster randomized control trial; classrooms randomly assigned to three conditions (building blocks; building blocks and scaffolding self-regulation; and business-as-usual control condition)	Cluster randomized trial; centers randomized to one of four conditions (tools of the mind, literacy express comprehensive preschool curriculum; combined curriculum; or business-as-usual condition)	Cluster randomized trial; centers randomized to tools of the mind condition or business-as-usual condition	Cluster randomized trial; centers randomly assigned to one of three curricula (IY, PP, or TM) or business-as usual control condition	Cluster-randomized trial; centers randomly assigned to tools of the mind condition or YMCA playing to learn curriculum condition	Block randomized trial; teachers were randomized into one of the three conditions (the self-regulation only version, the self-regulation plus math and reading version, or business-as-usual)
Sample location	Northeast of the United States	San Diego county	New Mexico and Massachusetts	2 southern states	17 Head Start grantees located in 10 states across the United States	Ontario, Canada	Pacific North West of the United States
Sample size	147 children	84 classrooms/teachers; 837 children	117 classrooms/teachers; 2,564 children	60 classrooms/teachers; 877 children	307 classrooms; 2,114 (total sample) and 678 tools-Play program group child sample	20 classrooms/teachers; 256 children	13 classrooms/teachers; 188 children
Child sample demographics	91% Hispanic with mean age of 5.1 years	39% Hispanic, Asian Pacific Islander 18%, African-American 11%, and 31% non-Hispanic White	52% Latino, 38% non-Latino with a mean age of 52.7 months	39% White; 29% Black; 24% Hispanic; 6% Asian, mean age 54.1 months (Tools sample); 41% White; 23% Black; 25% Hispanic; 6% Asian, mean age 54.6 months (Non-Tools sample)	43% were Hispanic, 33% African-American, and 16% White, with a mean age 4.49 years (full sample)	Study did not report the demographic information for children; mean age 45.9 months	58% Latino, 26% White, 7% Pacific Islander, 6% African American with a mean age of 51 months
**Intervention characteristics**							
Duration of implementation	2 years	2 years	2 years	2 years	1 year	2 years	8 weeks
Amount of training and coaching	7 days of training in year one; 2.5 days of training in year 2; coaching every 6 weeks	6 days of training in year 1 and 6 days in year 2 for tools; 6 days of training in Year 1 and 6 days in year 2 for the additional math curriculum; Biweekly coaching	Not reported	Not reported	5 training sessions; weekly coaching visits	5 training sessions in the first year and 2 training session in the second year; ongoing coaching	One half-day training; 6 coaching sessions
**Measures**							
Teacher practice					Adapted teacher style rating scale (TSRS) (OA)		
Classroom climate					CLASS (OA)		
Executive function and behavioral regulation	Dots Task (DA); Flanker (DA)	Forward and backward digit span (DA); HTKS (DA); Peg tapping (DA)	Behavior rating inventory of executive function-preschool version (TR); HTKS (DA)	Dimensional change card sort (DA); copy design (DA); corsi block-tapping task (DA); Peg tapping (DA); HTKS (DA); cooper-farran behavior rating scales (TR); Self-regulation assessor rating (AR)	Head-to-Toes (DA); pencil tap (DA); behavioral problem index (TR)	Day/Night task (DA); head-to-toes (DA)	HTKS (DA); day/night tasks (DA)
Emotion knowledge and social problem-solving skills					Facial emotions identification tasks (DA); emotion situation tasks (DA); challenging situations task (DA)		
Learning and social behaviors					Social skills rating system (TR)	Social competence and behavior evaluation scale (TR)	
Academic outcomes	One-Word-picture vocabulary (DA)		Bracken basic concepts scales-revised (DA); test of preschool early literacy (DA)	WJ-III Letter-word identification, spelling, oral comprehension, picture vocabulary, academic knowledge, applied problems, quantitative concepts (DA); adaptive language inventory (TR)	WJ-III letter-word identification (DA); WJ-III applied problems (DA); EOWPVT (DA); academic rating scale (TR)	Strengths and difficulties questionnaire (TR); early development index (TR); peabody picture vocabulary (DA); expressive vocabulary test (DA); get ready to read (DA); PTX (DA)	WJ-III letter-word identification (DA); preschool early numeracy screener (DA)

*DA, direct child assessment; TR, teacher-report; OA, observational assessment; AR, assessor-report*.

**Table 4:** Background characteristics of the cognitive skills studies.

**Study characteristics**	**Building Blocks Clements and Sarama, 2008**	**Building Blocks Hofer et al., 2013**	**Building Blocks–MPC study Morris et al., 2016; Mattera and Morris, 2017; Mattera et al., 2018**	**Building Blocks Clements et al., 2020**	**Building Blocks and OWL Weiland and Yoshikawa, 2013**
Design overview	Randomized control trial; classrooms were randomized to three conditions: building blocks; preschool mathematics curriculum; and business-as-usual	Cluster randomized control trial; preschools were randomly assigned to building blocks intervention or business-as-usual condition	Cluster randomized trial; preschool sites randomly assigned to building blocks intervention or business-as-usual control condition	Three-armed cluster randomized control trial; classrooms randomly assigned to three conditions (building blocks; building blocks and scaffolding self-regulation; and business-as-usual control condition)	Regression discontinuity design
Sample location		Buffalo, NY; Boston, MA; Nashville, TN	New York City, NY	San Diego county	Boston, MA
Sample size	35 teachers/classrooms and 253 children	139 classrooms/teachers and 1714 children	69 preschool sites, 173 classrooms/teachers; 1,389 children	84 classrooms/teachers; 837 children	250 teachers/classrooms; 2,018 children
Child sample demographics		Treatment condition: 17% White, 60% Black, 17% Hispanic, mean age 60 months; Control condition: 57% Black, 15% White, 22% Hispanic, with mean age 60 months	57% Hispanic, 3% Non-Hispanic White; 37% Non-Hispanic Black, 3% other, with mean age of 4.17 years	39% Hispanic, Asian Pacific Islander 18%, African-American 11%, and 31% non-Hispanic White	41% Hispanic, 26% Black, 18% White, 11% Asian, and 3% other
**Intervention characteristics**					
Duration of implementation	2 years	2 years	2 years	2 years	2 years
Amount of training and coaching	4 days and 2-hour refresher classes once every other month and monthly coaching	teacher training (7 days) and coaching	5 days of training in year 1 and year 2 (10 days total); weekly coaching	6 days of training in year 1 and year 2 and biweekly coaching	13 days of training; weekly to biweekly coaching
**Measures**					
Teacher practice	COEMET (OA)	COEMET (OA)	COEMET (OA)	COEMET (OA)	
Classroom climate			CLASS (OA)		
Executive function and behavioral regulation			Pencil tap (DA); spatial conflict arrows (DA); corsi blocks (DA)	Forward and backward digit span (DA); HTKS (DA); peg tapping (DA)	Forward digit span and backward digit span (DA); dimensional change card sort (DA); task orientation questionnaire (AR); pencil tap (DA)
Emotion knowledge and social problem-solving skills					Emotion recognition questionnaire (DA); TOQ positive emotion (TR); TOQ impulse control (TR)
**Learning and social behaviors**					
Academic outcomes	Early mathematics assessment (DA)	REMA (DA)	ECLS-B Math assessment (DA); WJ-III applied problems (DA); ROWPVT (DA)	Tools for early assessment of mathematics (DA); ECLS-B math (DA); EOWPVT (DA); renfrew bus story (DA); phonological awareness literacy screening (DA)	Peabody picture vocabulary test (DA); WJ-III applied problems and letter-word identification (DA); REMA (DA)

*DA, direct child assessment; TR, teacher-report; OA, observational assessment; AR, assessor-report*.

**Foundations of Learning (FOL)** combined teacher training in effective classroom management (Incredible Years Teacher Training Program, Webster-Stratton et al., 2004) with weekly classroom consultation (Morris et al., 2013). FOL rigorously tested this model by randomly assigning 71 centers to the program or a preschool-as-usual control condition in two sites (Chicago and Newark, NJ). One thousand two hundred three 4-year old children were assessed at the end of preschool. The study built upon an earlier smaller efficacy trial of the same multicomponent model (Chicago School Readiness Project; Raver et al., 2008, 2009).

**Head Start CARES** was a national, large-scale demonstration project explicitly designed to test three distinct approaches to enhancing children's social-emotional development in Head Start classrooms (Mattera et al., 2013; Morris et al., 2014). The three programs contrast alternative “levers for change” operating at different levels of the child's ecology. The study was a rigorous cluster-randomized controlled trial with over 100 preschools and 300 classrooms; 2,114 4-year-old children were assessed at the end of preschool. The three models tested were: The Incredible Years Teacher Training Program (IY), Preschool PATHS (Promoting Alternative Thinking Strategies; PATHS), and Tools of the Mind—Play (Tools-Play).

**Head Start REDI** (Research-based, Developmentally Informed) enriched Head Start classrooms with a social-emotional learning program (the Preschool PATHS Curriculum) and an interactive reading program, using daily small group reading sessions to reinforce PATHS social-emotional themes (Bierman et al., 2008). Weekly “sound games” and print center activities were also included, with the overall goal of promoting children's school readiness in areas of social-emotional learning, self-regulation, language development, and emergent literacy skills. Teachers received weekly classroom visits and meetings with REDI consultants to promote rich language use in the classroom and positive classroom management strategies. The program was evaluated in a randomized trial involving 44 preschool classrooms in 24 Head Start centers in three Pennsylvania counties assigned to intervention or “usual practice” comparison groups. Three hundred and fifty-six 4-year-old children were assessed at the start and end of the preschool year and then followed from prekindergarten through adolescence.

**Making Pre-K Count (MPC)** was a rigorous large-scale randomized controlled trial of an evidence-based preschool math curriculum (Building Blocks, Clements and Sarama, 2008) and professional development (Morris et al., 2016; Mattera et al., 2018). The MPC study was designed to experimentally test whether providing enhanced preschool math experiences for children could have positive effects not only on children's math skills, but on other domains of children's functioning, both in the short- and long-run. MPC took place in 69 pre-k sites, including 173 classrooms, across public schools or community-based organizations in New York City. Thirty-five sites were randomly assigned to receive the MPC intervention. Thirty-four sites continued with business-as-usual pre-k practices. One thousand, three hundred eighty-nine 4-year-olds were assessed in preschool as part of the study.

These trials attempted to promote EF with very different programmatic approaches. In the following sections, we describe the different approaches preschool programs have taken to improve EF and the evidence around the effectiveness of those approaches. Results of these studies are described along with other studies that have taken similar approaches.

## Approaches to School-Based School Readiness Interventions That May Promote EF

Theoretically, early flexible cognitive skills, and specifically EFs, may help bolster later academic knowledge (Blair, 2006). Accumulating research points to such early cognitive processes as important predictors of school readiness. Various facets of EF in the fall of the preschool year have been associated with math (Espy et al., 2004), verbal ability (Hughes, 1998; Blair and Razza, 2007), vocabulary, and literacy skills at the end of preschool (McClelland et al., 2007). The same relationship is replicated in later years; children with better learning behaviors in kindergarten have faster rates of growth in reading and math achievement through second grade, and differences in achievement between children with better and poorer learning behaviors are still evident in sixth grade (McClelland et al., 2006). Conversely, attention problems undermine effective learning, and contribute to off-task behavior, distractibility, and incomplete work, all contributing to learning delays and reduced achievement (Hughes and Kwok, 2006). Bi-directional influences are also evident, as growth in early academic skills in preschool contribute to growth in EF (Welsh et al., 2010; Clements et al., 2016).

Researchers have also hypothesized associations between early EFs and later social outcomes. Relations between executive dysfunction and poor social outcomes have been identified in such diverse groups as children with attention deficit hyperactivity disorder or conduct disorder, adults with traumatic brain injury, and preschoolers with disruptive behaviors (Pennington and Ozonoff, 1996; Hughes et al., 1998; Godfrey and Shum, 2000). Consequently, the relation between EF abilities and positive social outcomes has also been investigated. In elementary school children, EFs are positively associated with social competence and negatively predictive of problem behavior up to two years later (Riggs et al., 2006; Ciairano et al., 2007). Research with preschoolers has also found that EF is positively related to teacher reports of on-task behavior (Blair, 2002), delay of gratification (Carlson and Moses, 2001), and engaged learning behaviors (Brock et al., 2009). While EF and social-emotional skills do seem to be related, correlations are small and it is not clear whether EF skills are causally related to (that is, lead to) later social competence or whether the relationship is bi-directional (Sasser et al., 2015; Burchinal et al., 2020).

Associations between EF and school-related social and academic outcomes has made EF a promising additional target for school-based interventions. These programs have identified EF as a promising potential mechanism for subsequently or concurrently supporting social-emotional learning or academic outcomes. Those school-based interventions that identified EF as potential mechanism for improving school readiness fell under one of four types of approaches for improving children's skills and behaviors. While all of the approaches target domains of school readiness and teacher's practices in more than one way, each approach targets specific teacher practice or behavior pathways as a mechanism for targeting children's school readiness and, possibly, EF.

### Behavioral-Focused Interventions

Research has examined the relationship between the emotional and organizational climate of preschool contexts and children's self-regulation (Hamre and Pianta, 2005; Downer et al., 2010). Positive yet firm control in a classroom may provide a safe base to support children's learning and positive behaviors (Webster-Stratton et al., 2001). In behavioral-focused models, teachers set classroom routines, expectations, and discipline strategies that provide clear boundaries for children's behavior and learning. Children's relationships with teachers are key levers in this approach: teachers may help modulate children's behavior and emotions (Hoglund and Leadbeater, 2004; Chryssanthopoulou et al., 2005; Raver et al., 2007) with positive, supportive interactions; alternatively, in unregulated classrooms it has been found that teachers can reinforce children's dysregulation (Brouwers and Tomic, 2000). Through such cycles, teachers can either support or undermine children's behavioral self-regulation and, potentially, EF skills.

**Figure 1:**
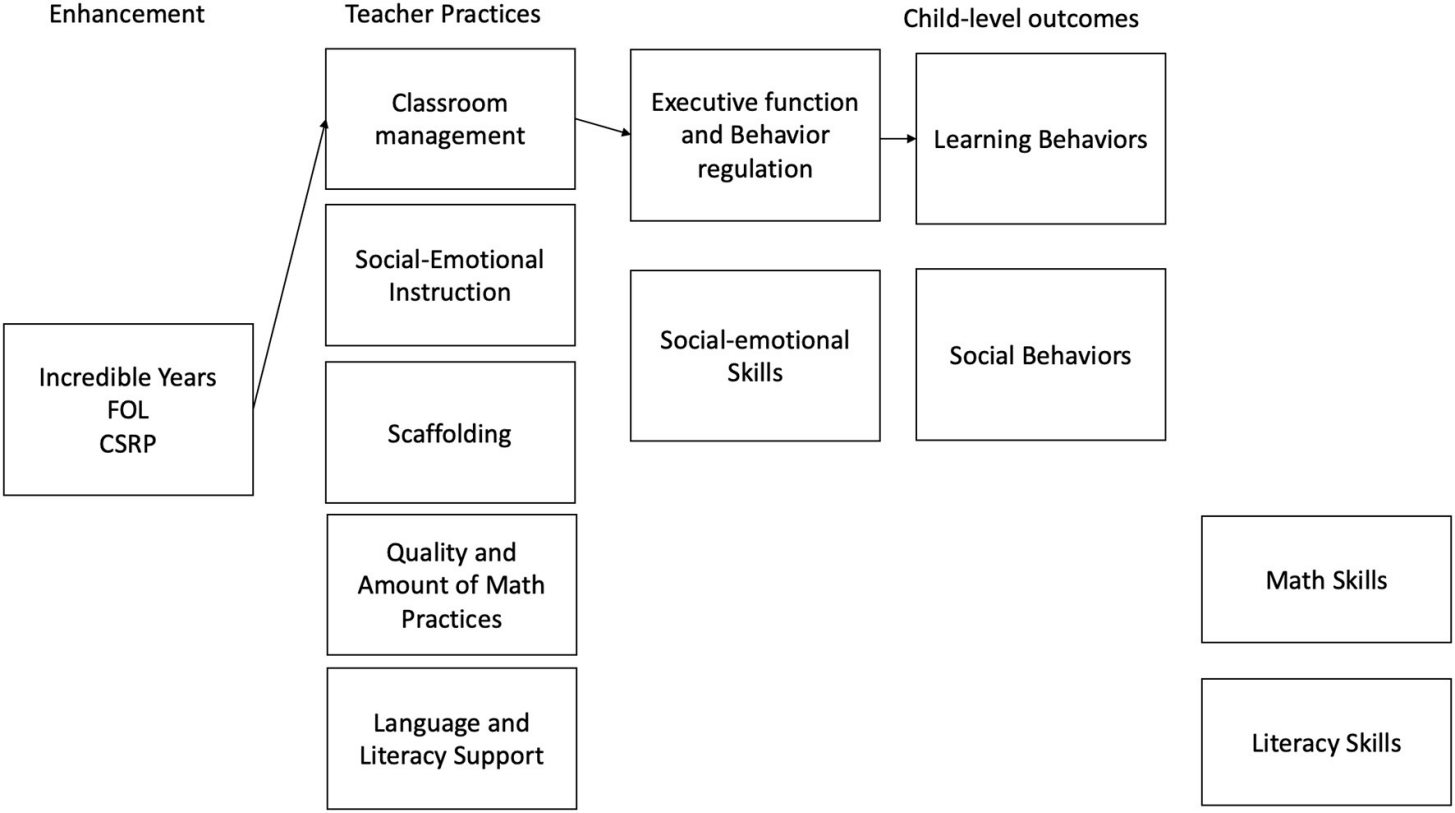
Hypothesized theory of change for behavioral-focused interventions.

Supporting teachers' ability to create a positive classroom climate through consistent and supportive limit-setting was one approach to improving children's behavioral and emotional regulation (Patterson et al., 1992; Webster-Stratton, 1998). The Incredible Years Teacher Training program is one example of this type of model that focuses on strengthening teacher classroom management, teacher-child relationships, and concrete strategies for supporting children's own emotional and behavioral regulation (Webster-Stratton et al., 2004), and potentially, EF. Figure 1 describes the hypothesized theory of change for the Incredible Years Teacher Training program.

Versions of the Incredible Years Teacher Training program alone have been tested in four rigorous randomized controlled trials with preschoolers (Webster-Stratton et al., 2004; Raver et al., 2009; Morris et al., 2013, 2014). Table 1 presents the details of each study, its design, sample intervention characteristics, and the measures used in the study. Incredible Years Teacher Training was found to have led to improvements in positive classroom management or organization, as expected, across all 4 trials. Secondarily, Foundations of Learning (FOL) found that Incredible Years led to improvements in classroom productivity and the amount of available instructional time, although not quality of instruction in the Newark site, where these outcomes were assessed (Morris et al., 2013). The program also led to the expected improvements in children's problem behaviors in the Chicago School Readiness Project (CSRP) and FOL-Newark and Webster-Stratton et al. (2004), but not in teacher reports of problem behaviors in FOL-Chicago or Head Start CARES. Incredible Years Teacher Training led to improvements in children's learning behaviors s in Head Start CARES, CSRP, and FOL-Newark, though not FOL-Chicago. Effects on positive social behaviors were mixed, with positive effects in Head Start CARES and Webster-Stratton et al. (2004) but not FOL. Effects on EF were also mixed, with positive effects in CSRP and FOL on EF and behavioral control, but not in the larger Head Start CARES trial. Despite effects on EF and social and learning behaviors in some trials, there were no effects of Incredible Years on children's early academic skills in FOL-Chicago or Head Start CARES, but CSRP did lead to improvements in children's pre-academic skills. Table 5 summarizes the pattern of effects on teacher and child outcomes across these studies of Incredible Years.

**Table 5:** The pattern of effects on teacher and child outcomes for behavioral-focused interventions.

	**The incredible years Webster-Stratton et al. (2004)**	**CSRP Raver et al., 2009**	**Foundations of learning–Newark Morris et al., 2013**	**Foundations of learning–Chicago Morris et al., 2013**	**The incredible years–CARES study Mattera et al., 2013; Morris et al., 2014**
**Teacher practice (observational assessment)**					
Classroom management	X				X
Social-emotional instruction					X
Scaffolding					0
Amount of math					
Math quality					
Literacy practices					0
**Classroom climate (observational assessment)**					
Classroom organization		X	X		0
Emotional support			X		0
Instructional support			0		0
**Executive function and behavioral regulation**					
Executive function		X		X	0
Behavior problems	X	X	X	0	0
**Emotion knowledge and social problem-solving skills (direct assessments)**					
Emotion knowledge					X
Social problem-solving skills				0	X
**Learning and social behaviors**					
Learning behaviors		X	X	0	X
Social behaviors	X		0	0	X
**Academic outcomes**					
Math		X		0	0
Language/Literacy		X		0	0

*In each cell, “X” indicates that there was a statistically significant impact on that outcome. The “0” indicates that the outcome was tested, and no positive statistically significant impact was found. The dark gray cells represent primary targeted outcomes for the program; the light gray cells represent secondary targeted outcomes; the white cells represent non-targeted outcomes. _FN_ represents a Kindergarten follow-up outcome*.

### Social-Emotional Learning Interventions

Another approach to improving children's social-emotional school readiness and, secondarily, EF, builds from social information processing theory (SIP; Crick and Dodge, 1994) and emotion theory (Izard, 2009). Social information processing theorizes that children's understanding of emotion and social problem-solving abilities help guide appropriate social responses to their peers (Hughes et al., 1998; Garner and Estep, 2001; Denham et al., 2003), and emotion theory posits that the capacity to identify and label different emotions helps guide effective emotion regulation and supports social interactions (Djambazova-Popordanoska, 2016). Evidence suggests that some children may have more difficulty in correctly identifying emotions in themselves and others or identifying socially acceptable solutions to social situations (Denham, 1997; Garner et al., 1997). A key lever in this theory involves strengthening children's explicit social-emotional skills to help them regulate their emotions and respond to challenging social situations appropriately, without misinterpreting social interactions or cues (Crick and Dodge, 1994; Raver and Spagnola, 2002). Improvements in these self-regulatory social and emotional abilities were also hypothesized to cooccur or even be underwritten by improvements in EF.

**Figure 2:**
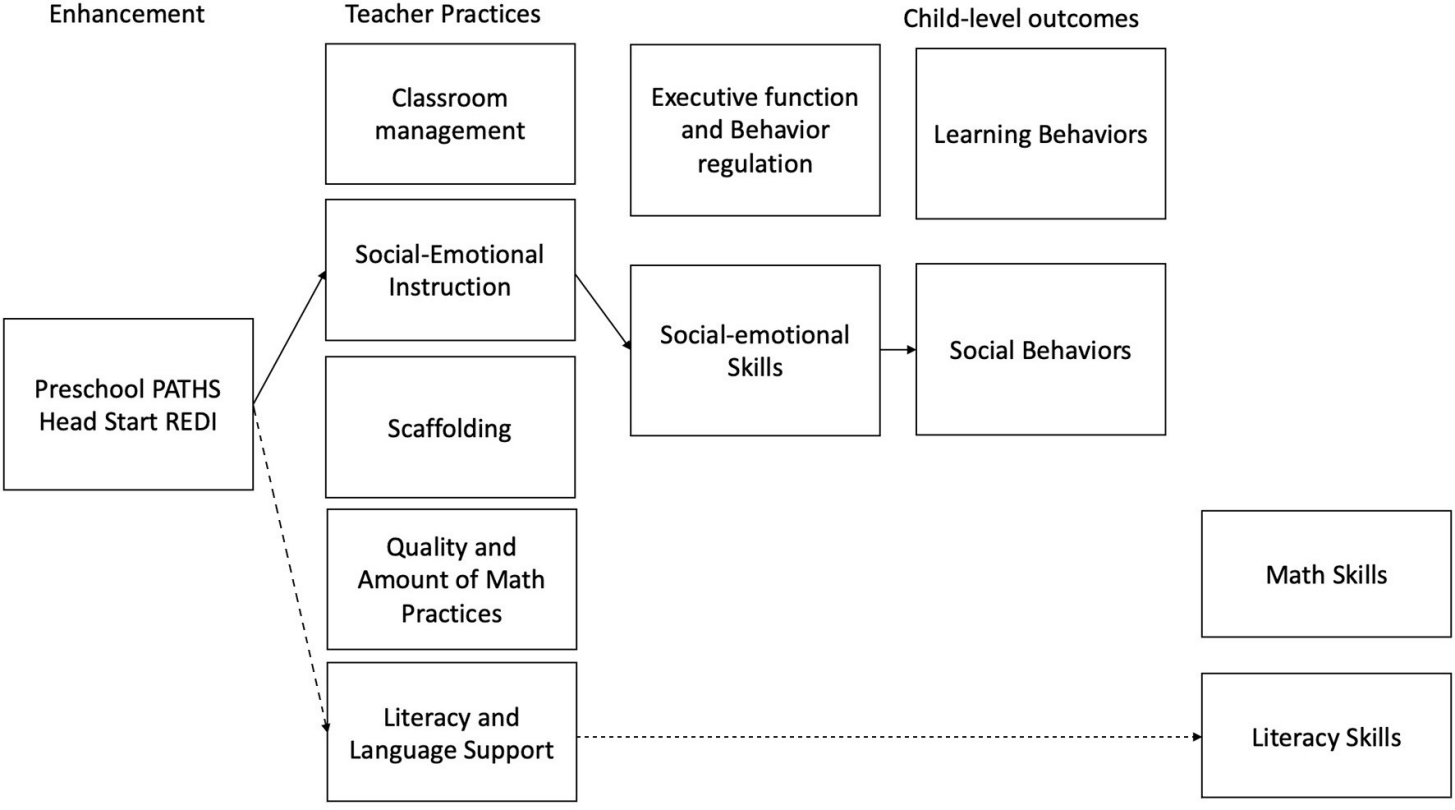
Hypothesized theory of change for social-emotional learning interventions.

Some preschool interventions have sought to support children's social and emotional development by explicitly teaching emotional knowledge and having children practice social problem-solving skills (Lynch et al., 2004; Domitrovich et al., 2007). Preschool PATHS (*P*romoting *A*lternative *Th*inking *S*trategies; Domitrovich et al., 2007) is one example of a program using this approach that uses a scripted curriculum to directly teach children about identifying emotions, strategies for self-regulation, and positive solutions to social situations. Teachers model and support children in naming a problem, using a self-regulation strategy, and practicing social problem-solving throughout the day. Figure 2 describes the hypothesized theory of change for the Preschool PATHS and Head Start REDI programs.

Preschool PATHS has been tested in rigorous trials with preschoolers as a stand alone curriculum (Domitrovich et al., 2007; Morris et al., 2014) and in combination with a language and literacy curriculum (Head Start REDI; Bierman et al., 2008). Table 2 presents the details of each study, its design, sample intervention characteristics, and the measures used in the study. PATHS improved teachers' use of instruction of social and emotional skills as expected (Bierman et al., 2008; Morris et al., 2014). Across all three trials, PATHS led to improvements on its targeted outcomes of emotion knowledge and teacher reports of social behavior in 2 of 3 trials. PATHS also led to improvements in children's teacher-reported learning behaviors in Head Start CARES and REDI. However, PATHS only led to improvements in EF when combined with a language and literacy program (Bierman et al., 2008), with no improvements in EF in the trials of the program alone. Similarly, PATHS only lead to improvements in children's literacy skills when combined with a literacy component in REDI. Table 6 summarizes the pattern of effects on teacher and child outcomes across these studies of Preschool PATHS.

**Table 6:** The pattern of effects on teacher and child outcomes across social-emotional learning interventions.

	**Preschool PATHS Domitrovich et al., 2007**	**Preschool PATHS–CARES study Mattera et al., 2013; Morris et al., 2014**	**Head Start REDI Bierman et al., 2008; Domitrovich et al., 2009**
**Teacher practice (observational assessment)**			
Classroom management		0	X
Social-emotional instruction		X	X
Scaffolding		0	
Amount of math			
Math quality			
Language and Literacy supports		0	X
**Classroom climate (observational assessment)**			
Classroom organization		0	0
Emotional support		0	0
Instructional support		X	0
**Executive function and behavioral regulation**			
Executive function	0	0	X
Behavior problems	0	0	X
**Emotion knowledge and social problem-solving skills (direct assessments)**			
Emotion knowledge	X	X	X
Social problem-solving skills	0	X	X
**Learning and social behaviors**			
Learning behaviors		X	X
Social behaviors	X	X	X
**Academic outcomes**			
Math		0	
Language/Literacy		0	X

*In each cell, “X” indicates that there was a statistically significant impact on that outcome. The “0” indicates that the outcome was tested, and no positive statistically significant impact was found. The dark gray cells represent primary targeted outcomes for the program; the light gray cells represents secondary targeted outcomes; the white cells represents non-targeted outcomes. _FN_ represents a Kindergarten follow-up outcome*.

### Interventions That Promote Play and Direct Training

Another approach more directly targets children's EF as a primary outcome of preschool intervention. This approach has children directly practice their EF skills, albeit in different ways.

**Figure 3:**
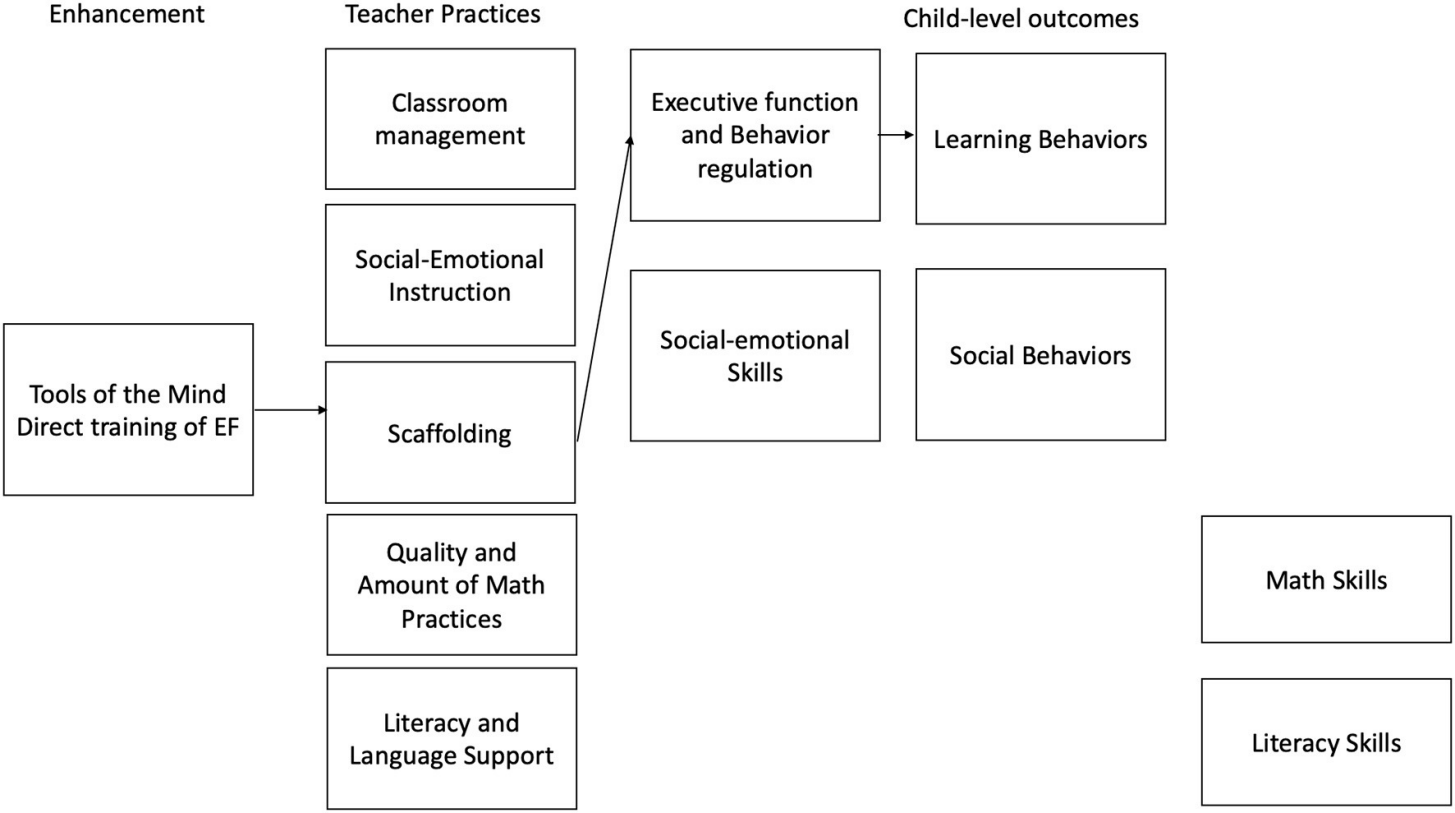
Hypothesized theory of change for promoting play and direct training interventions.

Tools of the Mind (Bodrova and Leong, 2007; Diamond et al., 2007) has children “practice” EF throughout the school day through tasks that require the use of self-regulation. Tools' central means of practicing EF expands on children's sociodramatic play as a mechanism for using working memory to plan play, shifting between multiple roles, and inhibiting other responses in role playing (Bodrova and Leong, 2009). The Tools approach is intended to help children learn to regulate their attention and behavior and interact positively with peers (Barnett et al., 2008). Tools also comprehensively reshapes the school day, creating larger blocks of pretend play time, having teachers support children's planning of their play extensively, and removing large amounts of whole group instruction. Figure 3 describes the hypothesized theory of change for the play components of the Tools of the Mind program.

A shortened version of Tools (Tools-Play; Morris et al., 2014) used in Head Start CARES improved teachers' scaffolding of pretend play, as expected. However, impacts on children's EF, the target of the Tools intervention, have been mixed. Six trials, described in greater detail in Table 3, have examined the effects of a version of Tools on preschool children's EF, with a smaller, earlier quasi-experimental study (Diamond et al., 2007) finding positive impacts on EF but five later randomized-controlled studies finding no evidence of program effects on EF (Clements et al., 2012; Lonigan and Phillips, 2012; Farran and Wilson, 2014; Morris et al., 2014; Solomon et al., 2018). Tools also had positive effects on children's math skills in 2 out of 5 trials where it was measured (Barnett et al., 2008; Morris et al., 2014), although the effects in the Head Start CARES trial were not corroborated across teacher reports and direct assessments. Table 7 summarizes the pattern of effects on teacher and child outcomes across these studies of Tools of the Mind.

**Table 7:** The pattern of effects on teacher and child outcomes across promoting play and direct training interventions.

	**Tools of the mind Diamond et al., 2007**	**Tools of the mind Clements et al., 2012**	**Tools of the mind Lonigan and Phillips, 2012**	**Tools of the mind Farran and Wilson, 2014**	**Tools of the mind–Play–CARES study Mattera et al., 2013; Morris et al., 2014**	**Tools of the mind Solomon et al., 2018**	**Red light/purple light McClelland et al., 2019**
**Teacher practice (observational assessment)**							
Classroom management					0		
Social-emotional instruction					0		
Scaffolding					X		
Amount of math							
Math quality							
Language and literacy supports					X		
**Classroom climate (observational assessment)**							
Classroom organization					0		
Emotional support					0		
Instructional support					0		
**Executive function and behavioral regulation**							
Executive function	X	0	0	0	0	0	X
Behavior problems					0	0	
**Emotion knowledge and social problem-solving skills (direct assessments)**							
Emotion knowledge					X		
Social problem-solving skills					0		
**Learning and social behaviors**							
Learning behaviors					0		
Social behaviors					0	0	
**Academic outcomes**							
Math			0	0	X	0	X
Language/Literacy			0	0	0	0	0

*In each cell, “X” indicates that there was a statistically significant impact on that outcome. The “0” indicates that the outcome was tested, and no positive statistically significant impact was found. The dark gray cells represent primary targeted outcomes for the program; the light gray cells represents secondary targeted outcomes; the white cells represents non-targeted outcomes. _FN_ represents a Kindergarten follow-up outcome*.

A different way of “practicing” EF comes from direct training programs [see, for e.g., Pauli-Pott et al. (2020), Takacs and Kassai (2019)]. These interventions provide children with repeated opportunities to train on EF exercises or tasks, theorizing that short-term improvements in performance on an EF task will generalize to more global improvements in self-regulation (Posner et al., 2006). Programs use either direct EF tasks (often in more lab-based settings) or EF games (for e.g., Red Light/Purple Light; McClelland et al., 2019) to achieve this purpose. Studies demonstrate that training on a specific EF task may lead to improvements in task performance (Klingberg et al., 2002; McClelland et al., 2019; Scionti et al., 2020). However, little evidence yet exists to support that these effects lead to the hypothesized impacts on more global social or learning behaviors in a school setting (McClelland et al., 2019).

Hybrid programs that use EF games in combination with other intervention strategies have also been tested. For example, the Second Step Early Learning curriculum combined a focus on lessons that target attentional control (e.g., listening, focusing attention, using self-talk to remember, and follow directions) and Brain Builder Games that practice EF skills with lessons that focused on broader social-emotional skills, such as empathy, emotion management, friendship skills, and social problem-solving skills (Upshur et al., 2017). A small randomized trial in Head Start classrooms documented significant effects on two EF measures, although only marginally significant effects emerged on the targeted social-emotional skills (Upshur et al., 2017).

### Improving Cognitive Skills Related to EF

A separate set of school readiness programs has also been found to have effects on children's EF. Unlike the other programs described above, these programs focus on children's cognitive or pre-academic skills (i.e., math and reading) instead of social-emotional development. While the main focus of these programs is on improving children's outcomes in cognitive domains, there is suggestive evidence that there may be spillover effects into EF.

**Figure 4:**
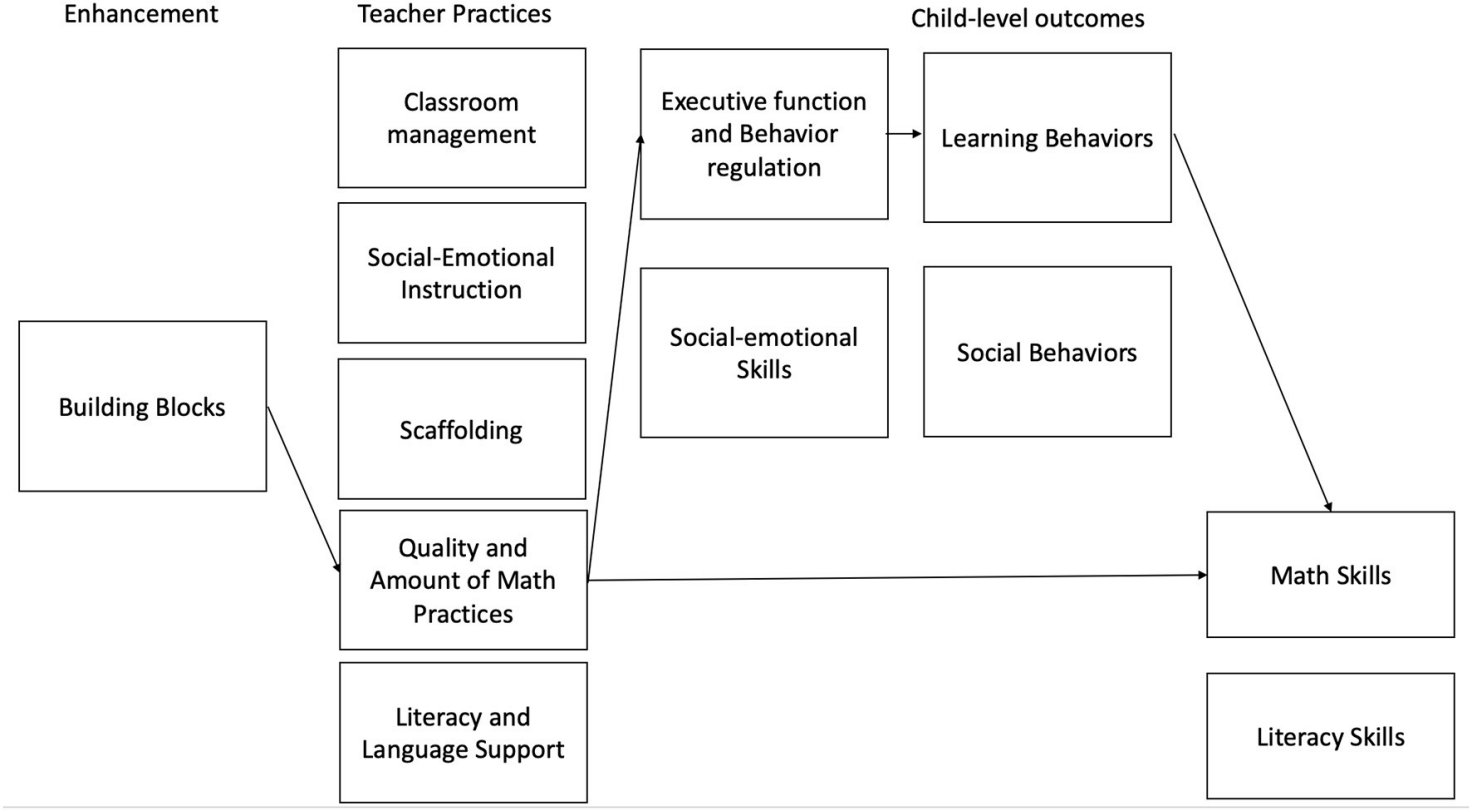
Hypothesized theory of change for cognitive skills interventions.

Math, for example, has been viewed as a way to improve a broad set of children's competencies in additional domains, including reading, and, although evidence is less strong, language, and EF.^2^ Short-term improvements in math have been hypothesized to spill over across domains of children's learning into language and EF. Math experts have suggested (albeit with somewhat limited empirical evidence) that engaging with math concepts like problem solving and sequencing skills may also support working memory and inhibitory control (Blair and Razza, 2007; Blair et al., 2008). Empirical work in this area has been quite limited until recently, but a few studies from pre-k math interventions (e.g., Weiland and Yoshikawa, 2013; Clements et al., 2020) as well as from correlational research (Blair and Razza, 2007) suggesting associations between math learning and EF. Figure 4 describes the hypothesized theory of change for the Building Blocks program.

The Building Blocks' math curriculum is an evidence-based preschool math curriculum with evidence of effects on children's math skills across five large-scale randomized controlled trials in 6 sites (Clements and Sarama, 2013; Hofer et al., 2013; Morris et al., 2016; Mattera et al., 2018; Clements et al., 2020) and a study of Building Blocks plus the Opening the World of Learning (OWL) literacy curriculum using a high-quality regression discontinuity design in Boston (Weiland and Yoshikawa, 2013). See Table 4 for further details about the trials. Building Blocks has been found to lead to more and higher quality math instruction in classrooms and various improvements in children's math in preschool (Clements and Sarama, 2013; Hofer et al., 2013; Weiland and Yoshikawa, 2013) or kindergarten (Mattera et al., 2018; Clements et al., 2020). Three studies have also examined whether the program might have spillover effects on children's EF. In all three studies, Building Blocks led to positive effects on children's EF in preschool, often that were sustained into kindergarten (Weiland and Yoshikawa, 2013; Morris et al., 2016; Mattera et al., 2018; Clements et al., 2020). Table 8 summarizes the pattern of effects on teacher and child outcomes across these studies of Building Blocks.

**Table 8:** The pattern of effects on teacher and child outcomes across cognitive skills interventions.

	**Building blocks Clements et al., 2011; Sarama et al., 2012**	**Building blocks Hofer et al., 2013**	**Building blocks–MPC study Morris et al., 2016; Mattera and Morris, 2017; Mattera et al., 2018**	**Building blocks Clements et al., 2020**	**Building blocks and OWL Weiland and Yoshikawa, 2013**
**Teacher practice (observational assessment)**					
Classroom management					
Social-emotional instruction					
Scaffolding					
Amount of math	X	X	X	X	
Math quality		X	X	X	
Language and literacy supports					
**Classroom climate (observational assessment)**					
Classroom organization			0		
Emotional support			0		
Instructional support			0		
**Executive function and behavioral regulation**					
Executive function			X	X	X
Behavior problems					
**Emotion knowledge and social problem-solving skills (direct assessments)**					
Emotion knowledge					X
Social problem-solving skills					
**Learning and social behaviors**					
Learning behaviors					
Social behaviors					
**Academic outcomes**					
Math	X	X	X FN	X FN	X
Language/Literacy			0	0	X

*In each cell, “X” indicates that there was a statistically significant impact on that outcome. The dark gray cells represent primary targeted outcomes for the program; the light gray cells represents secondary targeted outcomes; the white cells represents non-targeted outcomes. _FN_ represents a Kindergarten follow-up outcome*.

## Take-Aways

We draw four main takeaways for the field from these studies regarding the potential to improve children's EF.

**There Is Evidence That Some Preschool Approaches Do Result in Small Effects on EF, but Effects Vary by Type of Program and by Study**. Some studies of early childhood interventions focused on improving school readiness do find effects on EF. Effects on EF have been found in studies of teacher's behavior management (Raver et al., 2009; Morris et al., 2013) and direct instruction on non-social-emotional learning domains (i.e., math) (Weiland and Yoshikawa, 2013; Mattera et al., 2018; Clements et al., 2020). At the same time, the larger-scale, national Head Start CARES trial that was designed to explicitly test the effects of social-emotional learning approaches on preschool children's EF did not find effects of any of the tested approaches – behavior management, direct social-emotional learning instruction, or play/training-based approaches – on EF in preschool (Morris et al., 2014). Moreover, effects across studies that *do* find effects tend to be small, and it is hard as of yet to interpret what such effects mean in the long-term for children's outcomes. While the ECE research base in this area has not been able to make sufficient progress on impact variation to understand rigorously what is contributing to these inconsistencies, in laying out the pattern of effects across these trials, we hope to stimulate hypothesis generation and further empirical work on this question.

### Training Teachers Directly on Supporting EF in the Classroom Is Not the Only Way to Improve Children's EF

These studies show that it is possible to change teachers' behaviors in classrooms and improve children's social-emotional and self-regulatory outcomes, although these changes did not occur consistently across all studies. But perhaps somewhat surprisingly, training children directly in EF is not the only, or even perhaps the most effective way, to do so. Across the studies, a variety of teacher practices precipitated improvements in children's EF. For example, FOL (targeting teacher's behavior management strategies) led to improvements in teachers' classroom organization and children's EF; MPC (targeting math skills through lessons) led to improvements in teachers' math instruction and also children's EF. These changes in children's EF skills were tied to changes in the teachers' practices or in the classroom set up as a whole, not necessarily on directly teaching children explicit skills to support self-regulation or EF. Perhaps it is less about practicing EF in particular than about creating an environment that allows EF skills to develop.

### Measurement of EF Presents a Challenge for Studying the Effects of These Interventions on EF; Still, It Is Unlikely That the Lack of Consistent Impacts Is Solely Due to Measurement Issues Alone

One concern raised by experts in early childhood is the relatively gross level of measurement for children's EF. Measures used to assess EF in early childhood intervention studies range from measures of behavioral regulation (e.g., teacher reports of child behavior) to behavioral measures of cognitive skills (e.g., HTKS or Pencil Tap) to more specific computerized measures of cognition (e.g., Hearts and Flowers, Arrows). And, impacts on EF from preschool programs tend to cluster in certain types of measures – particularly behavioral measures of cognitive skills – rather than others (e.g., computerized cognitive measures). Over time, evidence has accumulated that measures of cognitive EF are not highly correlated with each other and with children's behavior or behavioral regulation in the classroom (Bierman et al., 2008; Morris et al., 2014). On the one hand, this suggests that how EF is measured across various ECE studies *could* influence the pattern of effects. On the other hand, despite the nascent and shifting characteristics of EF measures in early childhood, there are still signals that preschool programs can make headway in improving children's EF to some extent, suggesting that while measurement may play a role in whether impacts are observed, it cannot be the only reason for the lack or consistent effects. Clearly, what the field needs is a comprehensive and empirically-driven theory about how different EF measures perform in measuring program impacts and how they are associated with meaningful indicators of children's behavior and achievement in the classroom. Measures with greater reliability and developmental range along with more sensitivity to change could enhance the capacity to identify effects that occur.

### In the Current Research Base, It Is Difficult to Disentangle Issues of Measurement, Implementation Quality, and Sample Heterogeneity, From a Lack of Evidence on the Theoretical Drivers of EF

To ideally interpret this evidence base, all studies examining programs targeting EF in preschool would hold everything else constant, changing only the specific hypothesized driver of EF. However, studies and interventions vary in their sample, context, measurement, and the ability for the program to be implemented with high quality and fidelity. As described above, it is sometimes (though not entirely) difficult to disentangle whether a lack of impacts is due to theory, study methods, or other challenges with measurement.

## What's Next for the Field?

This paper summarizes a set of studies designed to test and strengthen the theory and evidence base about the possibility of improving EF in preschool children. As more findings have come out about these small but inconsistent effects, the field has stalled in developing new theories and subsequent interventions for bolstering EF. Our research over the past 15 years highlights additional questions that still remain to better establish a theoretical model of how and whether it is possible to identify preschool classroom approaches that consistently and meaningfully improve children's EF skills. Important questions about mechanisms and gaps in theory remain to be explored to better understand how and whether to target EF in young children.

### What Is the Right Timing of These Interventions?

Earlier neurodevelopmental research suggested that preschool was a promising time to intervene to support EF skills due to a rapid growth from age 3 to age 5 (Blair, 2006). However, findings from studies of programs targeting EF, culminating in Head Start CARES, find small and inconsistent effects on EF skills. In contrast, a number of meta-analyses and reviews of SEL programs in elementary school consistently find positive effects on social-emotional skills, including self-regulation (e.g., Durlak et al., 2011; Corcoran et al., 2018). A handful of studies have tested in elementary school some of the programs that were inconsistently related to EF in preschool. Both a study of Tools of the Mind in kindergarten (Blair et al., 2018) and PATHS in first grade (Riggs et al., 2006) find positive program effects on EF. Interestingly, neither of those programs have consistent evidence of effects on children's EF in preschool. It is possible these programs work more effectively on EF when children have more well-developed meta-cognitive skills to utilize the self-regulatory strategies more intentionally. No empirical work to our knowledge has directly examined whether effects on EF are larger depending on the timing of intervention relative to child age and developmental stage.

### What Drives Effects on Children's Outcomes?

Evidence across domains is accumulating that classroom processes and teacher practices are not as highly correlated with child outcomes as would be expected based on developmental theories and interventions (Burchinal, 2018). Similarly in the domain of EF, the evidence base to date does not clearly identify specific teacher practices that consistently are associated with changes in children's EF skills. A developing theory hypothesizes that it is the combination of teacher practices or processes *and* a base of content or curriculum that could lead to improvements in children's outcomes (McCormick et al., 2019). The studies described in this paper suggest that even changes in some targeted teacher practices might not be enough to improve children's EF, as in Tools of the Mind- Play (Morris et al., 2016). Instead, it is possible that content or curriculum in a specific domain are a useful vehicle for improving both teacher practices and subsequently children's EF (as in Building Blocks or REDI). Or, some integration with cognitive skill promotion could even be needed. Since EF skills are cognitive processes, it is possible that behavioral approaches are more effective when they also access and engage cognitive processes, as is done with more cognitive skill involvement.

One line of inquiry that has emerged in the wake of these studies is whether there are small, discrete teacher practices or packets of knowledge in the domain of EF that can be used without the need for a more complete EF curriculum [for e.g., Jones et al. (2017) description of “kernels”]. In other words, are there specific teacher instructional practices that can be identified, in the absence of curriculum, that directly improve children's EF? This theory posits that there are universal practices in a given domain that teachers can do to improve children's outcomes in that domain. However, the evidence base reviewed in this paper does not clearly identify if such kernels exist in the service of moving child outcomes in the domain of EF. Furthermore, an open question still remains about whether it is possible to change teachers' practice or knowledge in this way, disconnected from a curriculum.

### What Is the Relationship Between EF and Other Outcomes?

The evidence reviewed in this paper also suggests that more careful attention needs to be paid to explaining and defining the relationship between EF and other outcomes. Open questions remain about the added value of EF compared to classroom behavioral measures such as approaches to learning. Some studies examining similar EF-targeted programs have found program effects on EF, while others have found positive effects on approaches to learning. It is not clear why this might be and what these effects may mean for longer-term learning. Both the evidence and theory are similarly murky about the directionality of associations. Based on longitudinal research looking at early EF's relation with later outcomes, the presumption has been that improving EF first will promote cognitive and behavioral regulation second. But, several studies suggest that gains in academic skill influence subsequent EF growth (Welsh et al., 2010; Clements et al., 2016). And, other studies suggest that programs targeted at other domains of learning such as math may be equally as capable of moving EF, and also improve performance in other domains as well (Rabiner et al., 2010; Mattera et al., 2018; Clements et al., 2020). It is possible that learning new content and reasoning in new ways about literacy or mathematical challenges may promote EF growth as much as reflect it. Further research is needed to more rigorously disentangle the relationship between EF and regulation.

### What Role Does Scale Play in the Ability to Improve EF?

Many of the original studies that launched FOL, CARES, and MPC were smaller efficacy trials that worked closely with or were directly led by the developers of the interventions in one site or context. EF effects were found in many of these trials, in which levels of hands-on support of teachers was very high. In contrast, Head Start CARES scaled many of these programs up, testing them in over 300 classrooms around the country. In general, effects from interventions tend to be smaller and more diffuse when implemented on a larger scale (Wolf et al., 2020). While it is unclear why this is so, smaller focused studies run by developers may incorporate more implicit tailoring or adjusting the intervention approach to the specific location they are working, whereas larger studies generalize the approach across diverse settings.

It is also possible that different approaches may work more or less effectively in different contexts or with different subgroups of children. Many of the large-scale trials we describe in this article expanded the evaluation of these programs into other populations, including children from low-income families, racially or ethnically diverse samples, and various contexts (e.g., rural, urban). Variation in sample composition and children's individual differences may influence the pattern of effects from various evaluations. For example, some studies of programs targeting EF have found evidence of larger effects in children entering preschool with lower EF skills (Red Light, Purple Light and REDI; McClelland et al., 2019).

Twenty years ago, the early childhood intervention field sought to understand the promise and importance of EF for children's social and cognitive development. A set of rigorous trials have begun to unpack possible ways to support preschoolers' EF skills in the classroom, highlighting some approaches that have demonstrated success across multiple studies while identifying other approaches that have failed to consistently lead to improved EF. Yet, large questions remain for the field about the causal role EF plays in supporting later achievement and behavior, the appropriate timing for intervention, the teacher practices that support EF skills, and the measurement of EF.

## Author Contributions

SM wrote the manuscript. NR, PM, and KB revised the manuscript. All authors contributed to the article and approved the submitted version.

## Conflict of Interest

The authors declare that the research was conducted in the absence of any commercial or financial relationships that could be construed as a potential conflict of interest.
